# Prevalence of Medical Payment Products Promoted on US Hospitals’ Websites

**DOI:** 10.1001/jamahealthforum.2024.0231

**Published:** 2024-03-29

**Authors:** Samantha Randall, Nicholas Wong, Josephine Rohrer, Nina Linh Nguyen, Erin Trish, Erin L. Duffy

**Affiliations:** 1 Schaeffer Center for Health Policy and Economics, University of Southern California, Los Angeles; 2 Mann School of Pharmacy and Pharmaceutical Sciences, University of Southern California, Los Angeles

## Abstract

This cross-sectional study examines the prevalence of hospital-promoted medical payment products (MPPs) by whether hospitals offered any MPP or an interest-bearing MPP.

## Introduction

Fifty million individuals in the US have a financing plan to pay off medical or dental bills, with one-quarter of plans bearing interest.^1^ These plans are administered by hospitals, physician practices, or third-party companies. Medical payment products (MPPs) in the form of credit cards and loans by third-party financers have been scrutinized by the Consumer Financial Protection Bureau, the US Department of Health and Human Services, and the US Treasury because they may sidestep a range of patient and consumer protections and inflate medical bills with financing costs.^2^ MPPs are typically structured so that medical bills are paid upfront by the financer and patients pay off debt over time. MPPs may introduce consumer protections concerns when patients are marketed high-interest financing in lieu of aid or interest-free payment plans. This cross-sectional study describes the prevalence and characteristics of hospital-promoted MPPs, distinguishing those bearing interest, among a nationally representative sample of hospitals.

## Methods

The study sample comprised a 20% random sample of nongovernment, short-term general and surgical US hospitals as listed in the American Hospital Association (AHA) 2021 Annual Survey. Hospital websites were reviewed between June 1, 2023, and January 22, 2024, to identify MPPs and any other payment plans offered or promoted by the hospital. The University of Southern California institutional review board determined the study was not human participant research. This study followed the STROBE reporting guideline.

Hospitals were stratified by whether they offered any MPP or an interest-bearing MPP. Strata were compared on system affiliation, nonprofit vs for-profit ownership, graduate medical education (GME) affiliation, and number of hospital beds identified from the AHA Annual Survey^3^; Medicaid disproportionate share (DSH) status and total unreimbursed and uncompensated care cost as a percentage of operating expenses from RAND Hospital Data^4^; and states’ Medicaid expansion status.^5^ Analyses were conducted using Stata, version 17. Two-sample *t* tests compared continuous variables, and χ^2^ tests, binary categorical variables. Two-sided *P* < .05 was significant.

## Results

Of 670 hospitals in the sample, 129 (19.25%) offered any MPP. Hospitals offering any MPP were significantly more often nonprofit (96.12% vs 80.22%) and less often received Medicaid DSH payments (50.39% vs 65.62%) vs hospitals without any MPP (Table 1).

**Table 1.** Characteristics of Hospitals With and Without MPPs in a Nationally Representative 20% Sample of General and Surgical Hospitals

Characteristic	Hospitals^a^		*P* value
	With MPP (n = 129)	Without MPP (n = 541)	
System affiliated	105 (81.40)	417 (77.08)	.29^b^
Nonprofit ownership	124 (96.12)	434 (80.22)	<.001^b^
Graduate medical education affiliate	64 (49.61)	250 (46.21)	.49^b^
Receives Medicaid disproportionate share payment	65 (50.39)	355 (65.62)	.001^b^
Located in a state without Medicaid expansion	28 (21.71)	154 (28.47)	.12^b^
Total unreimbursed and uncompensated care cost, mean (SE), % of operating expenses^c^	6.85 (0.41)	7.09 (0.22)	.61^d^
Hospital beds, mean (SE), No.	191.8 (17.00)	188.2 (8.73)	.86^d^

Abbreviation: MPP, medical payment product.

^a^ Data are presented as number (percentage) of hospitals unless otherwise indicated.

^b^ Using χ^2^ test.

^c^ Year 2021 and includes Medicaid and Children’s Health Insurance Program shortfalls (costs less reimbursement), charity care, and bad debt. This measure is derived from hospital-reported data in the Centers for Medicare & Medicaid Services Healthcare Cost Report Information System and reported in RAND Hospital Data.^4^

^d^ Using 2-sample *t* test.

Only 57 hospitals (8.51% of total) promoted interest-bearing MPPs; 72 of the 129 hospitals (55.81%) promoting MPPs were not promoting interest-bearing products. Hospitals offering interest-bearing MPPs were significantly more often nonprofit (94.74% vs 82.22%) and had higher total 2021 unreimbursed and uncompensated care costs as a percentage of operating expenses (8.51% vs 6.90%) than other hospitals (Table 2). Although not statistically significant, hospitals promoting interest-bearing MPPs disproportionately had fewer beds (154.3 vs 192.1) and were less likely to receive a Medicaid DSH payment (52.63% vs 63.62%). GME affiliation, system membership, and state Medicaid expansion did not meaningfully differ.

**Table 2.** Characteristics of Hospitals With and Without Interest-Bearing MPPs in a Nationally Representative 20% Sample of General and Surgical Hospitals

Characteristic	Hospitals^a^		*P* value
	With interest-bearing MPP (n = 57)	Without interest-bearing MPP (n = 613)^b^	
System affiliated	44 (77.19)	478 (77.98)	.89^c^
Nonprofit ownership	54 (94.74)	504 (82.22)	.02^c^
Graduate medical education affiliate	26 (45.61)	288 (46.98)	.84^c^
Receives Medicaid disproportionate share payment	30 (52.63)	390 (63.62)	.10^c^
Located in a state without Medicaid expansion	18 (31.58)	164 (26.75)	.43^c^
Total unreimbursed and uncompensated care cost, mean (SE), % of operating expenses^d^	8.51 (0.71)	6.90 (0.20)	.02^e^
Hospital beds, mean (SE), No.	154.3 (21.62)	192.1 (8.24)	.17^e^

Abbreviation: MPP, medical payment product.

^a^ Data are presented at number (percentage) of hospitals unless otherwise indicated.

^b^ Hospitals without interest-bearing MPPs either did not promote an MPP (n = 541) or promoted an MPP that did not accrue interest (n = 72).

^c^ Using χ^2^ test.

^d^ Year 2021 and includes Medicaid and Children’s Health Insurance Program shortfalls (costs less reimbursement), charity care, and bad debt. This measure is derived from hospital-reported data in the Centers for Medicare & Medicaid Services Healthcare Cost Report Information System and reported in RAND Hospital Data.^4^

^e^ Using 2-sample *t* test.

Hospital-administered payment plans were offered by 583 hospitals (87.01%). Plan details were available online for 150 (22.39%) of these; the remainder provided a telephone number. Among those with detail online, 4 explicitly charged interest or fees (5.26% and 9% annual percentage rate; $3.95/mo and $4.95/mo). Seventy-two hospitals (10.75%) offered neither an MPP nor a payment plan.

## Discussion

This study identified interest-bearing MPPs offered on 8.51% of hospitals’ websites. Nonprofit hospitals and those with more unreimbursed and uncompensated care disproportionately promoted interest-bearing MPPs, possibly to mitigate their financial strain. These findings suggest that policies limiting hospitals’ promotion of MPPs may need to be coupled with efforts to stabilize hospital finances, as hospitals may be using MPP promotion when they lack the resources to offer long-term interest-free financing. Potential regulations should target specific aspects of product design rather than third-party medical financing broadly. This study is limited by using a 20% sample of hospitals, the small number of MPPs, and challenges accessing hospitals’ full payment options and details without a patient portal account.

## Notes

Supplement. Data Sharing Statement
