# Supplementary material for: Prevalence of Medical Payment Products Promoted on US Hospitals’ Websites
Source: JAMA Health Forum. 2024 Mar 29;5(3):e240231. doi: 10.1001/jamahealthforum.2024.0231 (PMC10980956; doi:10.1001/jamahealthforum.2024.0231)
Supplement: Supplement. — Data Sharing Statement [file jamahealthforum-e240231-s001.pdf]

Data Sharing Statement

Randall. Prevalence of Medical Payment Products Promoted on US Hospitals' Websites. *JAMA Health Forum*. Published March 29, 2024. doi:10.1001/jamahealthforum.2024.0231

Data
Data available: No
